# Larger omental adipocytes correlate with greater Fetuin-A reduction following sleeve gastrectomy

**DOI:** 10.1186/s40608-019-0238-4

**Published:** 2019-05-06

**Authors:** Katie N. Robinson, Blair Rowitz, Uretz J. Oliphant, Sharon M. Donovan, Margarita Teran-Garcia

**Affiliations:** 1 Department of Internal Medicine, University of Iowa, Iowa City, IA USA; 2 Carle Illinois College of Medicine, Urbana, IL USA; 3 Department of Surgery, Carle Foundation Hospital, Urbana, IL USA; 4 Division of Nutritional Sciences, University of Illinois at Urbana-Champaign, Urbana, IL USA; 5 Department of Food Science and Human Nutrition, University of Illinois at Urbana-Champaign, Urbana, IL USA; 6 Department of Human Development and Family Studies, University of Illinois at Urbana-Champaign, Urbana, IL USA

**Keywords:** Fetuin-a, Sleeve gastrectomy, Preoperative diet

## Abstract

**Background:**

Shortly after bariatric surgery, insulin sensitivity improves and circulating Fetuin-A (FetA) declines. Elevated FetA may decrease insulin sensitivity by inhibiting insulin receptor autophosphorylation. FetA also mediates inflammation through toll-like receptor 4 and influences monocyte migration and macrophage polarization in the adipocyte. The role of dietary changes on FetA is unclear. It is also unknown whether changes in FetA are associated with adipocyte size, an indicator of insulin sensitivity.

**Methods:**

Sleeve gastrectomy patients (*n* = 39) were evaluated prior to the preoperative diet, on the day of surgery (DOS) and six-weeks postoperatively. At each visit, diet records, anthropometrics and fasting blood were collected. Adipocyte diameter was measured in omental adipose collected during surgery.

**Results:**

Although significant weight loss did not occur during the preoperative diet, HOMA-IR improved (*p* < 0.0001) and FetA decreased by 12% (*p* = 0.01). Six-weeks postoperatively, patients lost 9% of body weight (*p* = 0.02) and FetA decreased an additional 26% (*p* < 0.0001). HOMA-IR was unchanged during this time. Omental adipocyte size on DOS was not associated with preoperative changes in dietary intake, body composition or HOMA-IR. However, adipocyte size was strongly associated with both pre- (*r* = 0.41, *p* = 0.03) and postoperative (*r* = − 0.44, *p* = 0.02) change in FetA.

**Conclusion:**

FetA began to decrease during the preoperative diet. Greater FetA reduction during this time was associated with smaller adipocytes on DOS. Therefore, immediate, post-bariatric improvements in glucose homeostasis may be partly explained by dietary changes. The preoperative diet protocol significantly reduced insulin resistance, a modifiable risk factor for other non-bariatric procedures. Therefore, this dietary protocol may also be used preoperatively for procedures beyond bariatric surgery.

## Background

Bariatric procedures result in significant weight loss and resolution of obesity-associated diseases. In 2013, 468,609 patients with morbid obesity opted for surgical treatment of obesity. The procedure that is growing most rapidly in popularity is the sleeve gastrectomy (SG) which accounted for 37% of all operations [1]. On average, SG patients lose 62.3% of excess body weight 5 years after surgery and resolution rates for Type 2 Diabetes Mellitus (T2DM) are near 80%. Thus, important insights about the pathophysiology of T2DM may be gained from understanding the mechanisms behind SG outcomes [2–5].

The hepatokine, Fetuin-A (FetA), reduces significantly after bariatric surgery, consequently, it has emerged as a potential novel marker of glucose homeostasis improvements. FetA has been suggested to play a role in glucose metabolism through two mechanisms: 1) FetA inhibits insulin receptor autophosphorylation, therefore, limiting translocation of the glucose transporter 4 and 2) when bound to saturated fatty acids, FetA can signal the inflammatory cascade through toll-like receptor 4, thus promoting insulin resistance [6, 7]. Elevated FetA has been linked to adverse health conditions including T2DM and cardiovascular disease [8–10]. Therefore, postsurgical reductions in FetA may partially explain the immediate improvements in glucose homeostasis following bariatric procedures.

To date, three studies have evaluated change in FetA following weight loss surgery. Although study lengths and protocols varied, all found that postoperative FetA was significantly lower than preoperative FetA. In a metabolomic analysis, FetA was one protein to significantly decrease 3 days after Roux-en-Y Gastric Bypass (RYGB) when compared to 3 days before surgery. In this short time period, plasma FetA decreased by 27% in the absence of significant weight loss [11]. FetA was also significantly reduced 1 year after RYGB, mini-gastric bypass, and SG. Overall, the average change was − 10% but the largest decrease (near − 14%) was seen in the group receiving SG [12]. Sixteen months after RYGB, average BMI was decreased by 34% and FetA was decreased by 19% [13]. These studies suggest that bariatric procedures result in the rapid and sustained reduction of FetA. It is unclear whether this reduction in FetA is unique to surgery or can be partially explained by dietary restriction.

Circulating FetA responds to dietary changes. Exposure to high-fat diets increased FetA in vitro and in animal models [14]. Hepatocytes exposed to saturated fatty acids; specifically palmitate, secreted FetA in a dose-dependent manner and had higher expression of FetA mRNA [15]. FetA is also reduced by caloric restriction [16]. Thus, when attempting to study the response of FetA to surgery, it is important to consider the role of the diet.

In the weeks preceding bariatric surgery, patients are often prescribed a hypocaloric, low-fat diet. The preoperative diet is recommended to decrease liver size and improve access to the stomach and gastro-esophageal junction [17]. Of the studies mentioned previously, only one [11] reported the timing of the initial FetA measurement and none reported whether preoperative diets were completed. Thus, the reported baseline FetA levels may have already been influenced by preoperative diet regimen. Therefore, our objective was to describe changes in FetA during the preoperative diet and during the weeks immediately following SG. Because adipocyte size has been positively correlated with body fat percentage and insulin resistance, we also evaluated whether circulating FetA change was correlated with omental adipocyte size [18, 19].

## Methods

### Data collection

Forty-five SG patients were recruited from Carle Foundation Hospital (Urbana, IL). Patients were excluded if they were current smokers, < 18 years old or had a pacemaker or implanted defibrillator. Participants attended three visits; baseline (T0) took place 2 weeks before surgery (median: 18 days, mode: 15 days). The second appointment (T1) occurred following the two-week preoperative diet on the morning of surgery. The final appointment (T2) occurred six-weeks following surgery (median: 41 days, mode: 41 days). The Individual Outcomes of Weight Loss Surgery (I-OWLS) Study protocol was approved by Carle Foundation Hospital and University of Illinois at Urbana-Champaign (UIUC) Institutional Review Boards.

A Registered Dietitian advised each patient on the pre- and postoperative dietary protocol. During the 2 weeks before surgery, men were advised to consume 1000 cal and 70–90 g of protein per day. Women were advised to consume 800 cal and 50–60 g of protein per day. All patients were encouraged to drink 64 oz of water each day and to split daily calories into six small meals including two to three servings of the following: fruits, vegetables, dairy and oral protein supplements. Example meal plans were provided to each participant. The postoperative diet was a standard transitional diet: full liquid for postoperative weeks 1–2, pureed diet for postoperative weeks 3–4 and regular foods during postoperative week 5. Three-day food logs (including two weekdays and one weekend day) were collected at each appointment to assess dietary adherence. Food logs were analyzed using the Nutrition Data System for Research software developed at the University of Minnesota [20].

Participants were asked to arrive at each appointment fasted, to avoid alcohol for 24 h prior to the visit and to avoid exercise for 12 h prior to the visit. Height, weight and body composition (InBody230, Biospace, Cerritos, CA) were measured by trained research staff (K.R.). Laparoscopic SG was performed by one of two surgeons (B.R. and U.O.) at Carle Hospital following previously established protocols. During surgery, 1–2 g of adipose tissue was collected from the omentum along the greater curvature of the resected stomach.

### Blood analysis

Fasting blood was collected by trained phlebotomists at Carle Hospital. Blood was centrifuged within 30 min at 10,000 g for 10 min, separated into aliquots and stored at − 80 °C until further analysis. Plasma FetA and insulin were measured by enzyme-linked immunosorbent assays (BioVendor™, Asheville, NC and EMD Millipore, Billerica, MA, respectively). Blood lipids were measured by LabCorps (Dublin, OH) and glucose was measured in duplicate by glucometer (Trividia Health, Fort Lauderdale, FL). Homeostatic Model Assessment for Insulin Resistance (HOMA-IR) was calculated using Matthews’ equation [21].

### Tissue histology

Omental adipose tissue was available from 32 participants. Formalin-fixed and paraffin-embedded tissues were cut into 5 μm slices, mounted on slides and stained with hematoxylin and eosin by the Comparative Biosciences Histology Laboratory at the College of Veterinary Medicine at UIUC. Slides were scanned at 20x by Nanozoomer slide scanning system (Hamatsu, Hamamatsu City, Japan) to capture images. Adipocyte area and diameter was calculated using Adiposoft software plugin for Image J (NIH, Bethesda, MD). Following analysis in automated mode, each individual image was evaluated for improper gating. Adipocytes that touched the field border were excluded from analysis. Average adipocyte diameter and area were calculated from 100 randomly selected adipocytes from three fields within each individual tissue sample. Participants were not asked to alter current medication use during the duration of this study. Thus, those taking Thiazolidinediones (TZDs), which alter peroxisome proliferator-activated receptor gamma (PPAR-γ), and, thus, may alter adipocyte size, were excluded from analysis (*n* = 3).

### Statistics

Variables were evaluated for errors and normality. Non-normal variables were transformed for analysis and back-transformed for interpretation. The effect of preoperative diet and SG on FetA was assessed by calculating the change between T0 and T1 and between T1 and T2 appointments, respectively. For longitudinal dietary analysis, participants with complete diet logs from all three appointments were included (*n* = 32). Ideal body weight was estimated based on a reference BMI of 25 kg/m^2^ and used to calculate excess weight (EBW) at baseline and EBW loss at T1 and T2. Individuals were categorized as insulin resistant if they had a documented diagnosis of prediabetes or diabetes, a HOMA-IR greater than 2.5, and/or a current prescription for anti-diabetic medications. If they did not meet any of these criteria, they were categorized as insulin sensitive. During comparisons of individuals with insulin sensitive and insulin resistance, one extreme outlier was excluded. Time was treated as a within-subject factor. Thus, repeated measures of time were considered. Differences between time points were compared using mixed models and contrast statements as appropriate. Because age has been found to be correlated with FetA, models were adjusted for age (in years) when appropriate [22]. Data is presented as mean ± SD unless otherwise indicated. All statistical analysis was performed in SAS 9.4 (Cary, NC).

## Results

### Demographics

**Fig. 1:**
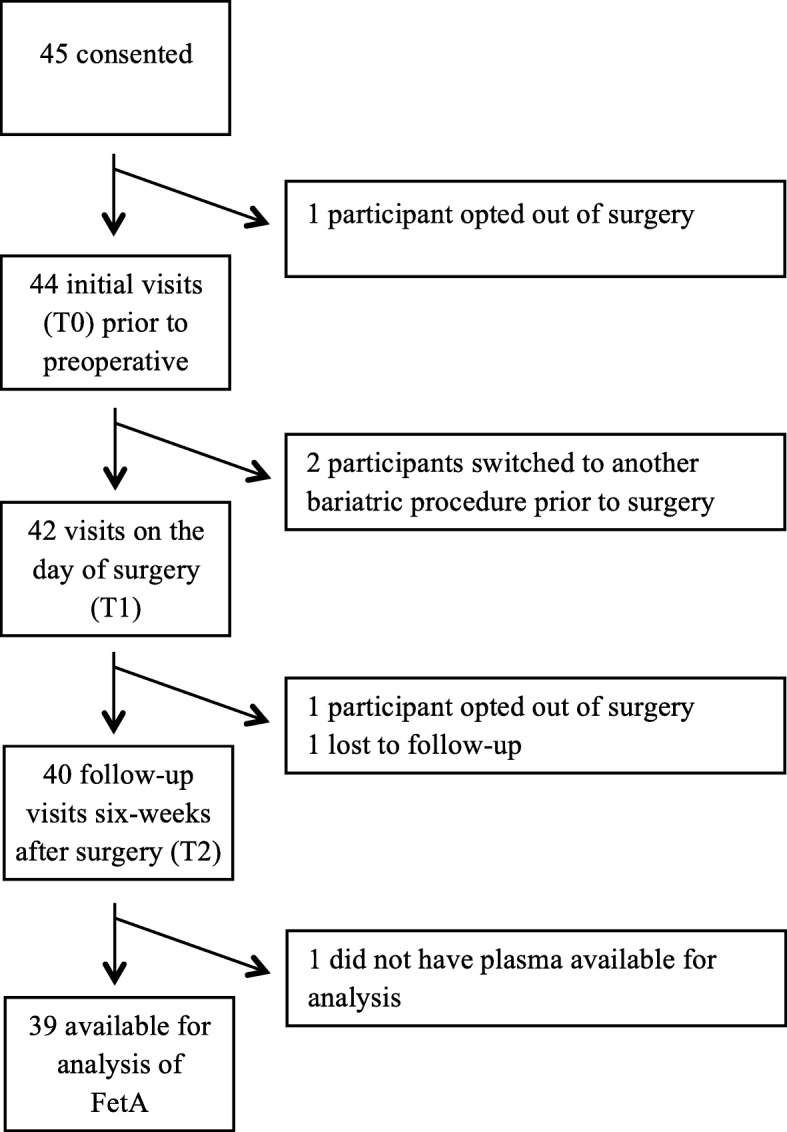
Participant flow diagram for the I-OWLS cohort. Participants were recruited June 2015 through March 2016. Of the 45 individuals who were consented for the study, 89% completed all three scheduled appointments

The study protocol was completed by 40 participants (77.5% female) (Fig. 1, Table 1), of whom 85% were non-Hispanic white and 15% were non-Hispanic black. At baseline, average BMI was near 47 kg/m^2^ for both genders and baseline percent body fat (% BF) was 52% for females and 47% for males. Of the cohort, 66.7% had either a HOMA-IR greater than 2.5, a documented diagnosis of prediabetes or diabetes and/or a current prescription for anti-diabetic medications. Baseline average calorie intake (±SE) was 1730 ± 104 cal/day with a macronutrient distribution of 43% carbohydrate, 19% protein and 38% fat. When stratified by sex, the only significant difference was greater (*p* = 0.05) fat consumption in men (42 ± 7.3%) when compared to women (37 ± 6.3%).

**Table 1:** Baseline characteristics by insulin sensitivity status

Variable	Insulin Resistant	Insulin Sensitive	*p*-value
N	26	13	
Female (%)	76.9	76.9	
Age (years)	43.7 ± 2.2	44.1 ± 1.6	0.92
BMI (kg/m^2^)	47.7 ± 1.5	46.2 ± 1.4	0.54
Percent Body Fat	50.5 ± 1.0	51.4 ± 0.6	0.55
TG (mg/dL)	152.5 ± 16.1	140.0 ± 15.9	0.63
TC (mg/dL)	166.1 ± 6.1	194.6 ± 12.5	0.02
HDL (mg/dL)	36.1 ± 1.9	50.0 ± 3.4	0.0007
LDL (mg/dL)	101.2 ± 6.1	116.7 ± 10.5	0.18
Glucose (mg/dL)	108.9 ± 3.6	96.1 ± 1.6	0.03
HOMA-IR	3.8 ± 0.4	1.5 ± 0.1	< 0.0001
Fetuin-A (μg/mL)	575.9 ± 32.6	666.5 ± 46.3	0.11

Values are presented as means ± SE. *p*-values less than 0.05 are considered statistically significant. Abbreviations: *BMI* Body Mass Index, *TG* Triglycerides, *TC* Total Cholesterol, *LDL* Low Density Lipoprotein, *HDL* High Density Lipoprotein, *HOMA-IR* Homeostatic Model Assessment for Insulin Resistance

### Caloric intake, excess weight and HOMA-IR were significantly reduced during the pre- and postoperative period

**Fig. 2:**
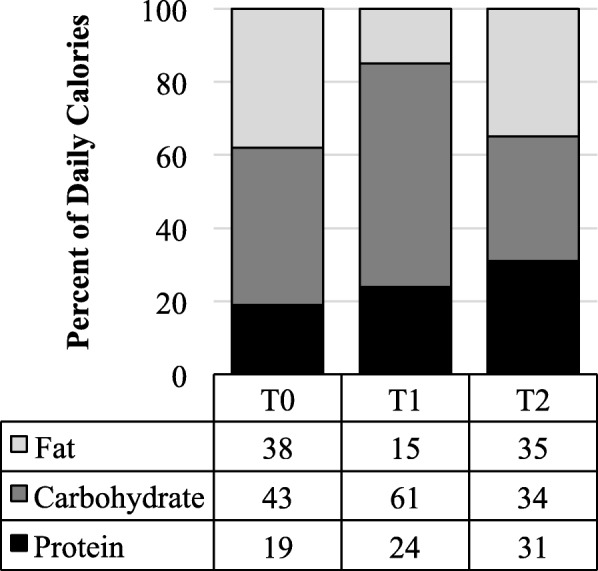
Reported macronutrient distribution range of sleeve gastrectomy patient by visit. Three-day dietary records were collected from participants at each visit. Logs were analyzed in Nutrition Data System for Research software. Macronutrient distribution was calculated by dividing average total calories of fat, protein and carbohydrate by average daily caloric intake

Dietary intake differed (*p* < 0.0001) across time points. During the preoperative and postoperative diet, intake (±SE) decreased to 960 ± 45 and 735 ± 49 cal/day, respectively. No sex differences were found in reported dietary intake at either T1 or T2. Macronutrient distribution of the preoperative diet was 61% carbohydrate, 24% protein and 15% fat whereas distribution at T2 was 34% carbohydrate, 31% protein and 35% fat (Fig. 2).

Significant weight change was observed during the study. Weight loss during the postoperative period was greater than preoperatively. Following the preoperative diet, men lost 10.8 ± 3.3% of EBW while women lost 9.9 ± 4.6% of EBW. On average, postoperative EBW loss was 19.5 ± 8.5% in men and 18.2 ± 6.9% in women. Weight change did not differ between those with insulin sensitivity and those with insulin resistance (Table 2). During the preoperative diet, significant improvements in fasting HOMA-IR were noted (*p* < 0.0001). At T0, average HOMA-IR (±SE) was 2.75 ± 0.26 and at T1, HOMA-IR was 1.20 ± 0.11. At T2, HOMA-IR was not lower than on the morning of surgery (1.17 ± 0.10, *p* = 0.847).

**Table 2:** Pre- and postoperative weight loss by insulin sensitivity status

Variable	Insulin Resistant	Insulin Sensitive	*p*-value
N	26	13	
Preoperative			
Change in Fetuin-A	−47.7 ± 30.4	− 122.9 ± 52.3	0.19
Change in BMI (kg/m^2^)	−2.2 ± 0.2	−2.1 ± 0.2	0.64
Change in Percent Body Fat	− 0.4 ± 0.3	− 0.4 ± 0.3	0.12
Excess Body Weight Loss	−10.4 ± 1.0	− 9.9 ± 0.8	0.76
Postoperative			
Change in Fetuin-A	− 158.9 ± 21.4	−104.9 ± 25.8	0.13
Change in BMI (kg/m^2^)	−4.4 ± 0.3	− 3.4 ± 0.3	0.07
Change in Percent Body Fat	−2.5 ± 0.3	−1.9 ± 0.2	0.26
Excess Body Weight Loss	−19.6 ± 1.4	−17.3 ± 2.0	0.35

Values are presented as means ± SE. *P*-values less than 0.05 are considered statistically significant. Abbreviations: *BMI* Body Mass Index

### Response of circulating FetA to the preoperative diet and sleeve gastrectomy

**Fig. 3:**
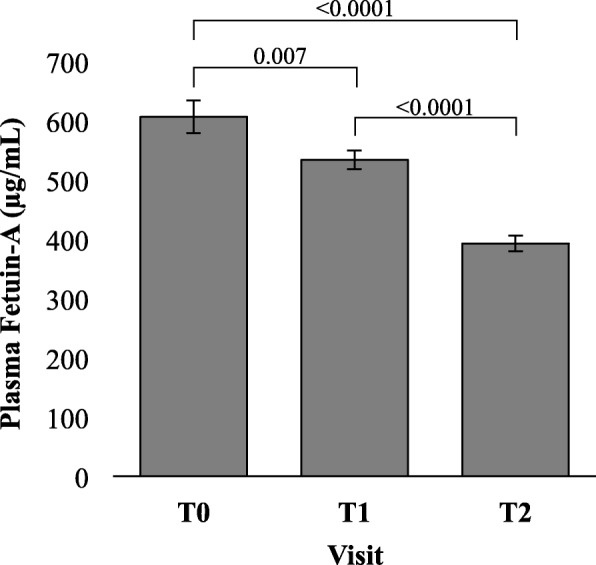
Fetuin-A in I-OWLS patients at baseline (T0), on the morning of surgery (T1) and at six-week follow-up (T2). Fasting plasma Fetuin-A was significantly reduced on the morning of surgery and at six-week follow-up compared to baseline. Data are adjusted for age and presented as mean ± SE

**Fig. 4:**
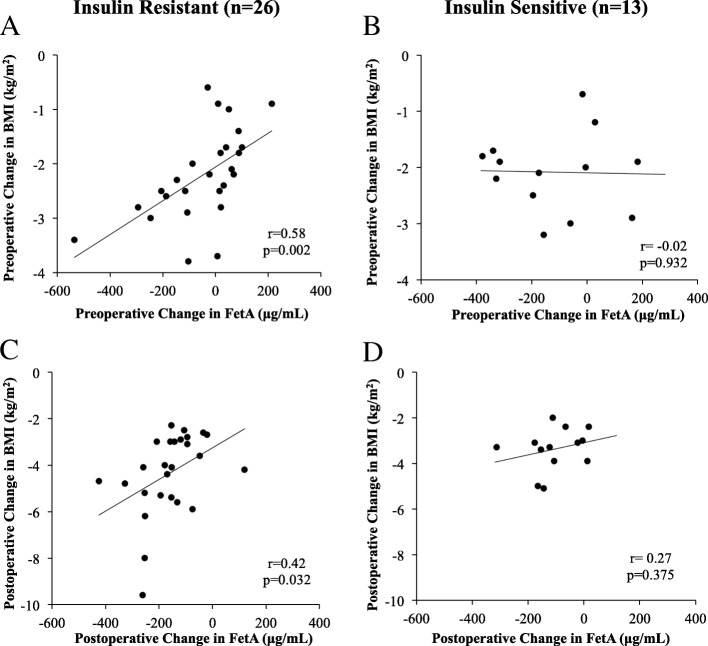
Correlation of Fetuin-A change with body mass index (BMI) change during the pre- and postoperative period by insulin sensitivity status; **a** preoperative correlation in individuals with insulin resistance; **b** preoperative correlation in individuals with insulin sensitivity; **c** postoperative correlation in individuals with insulin resistance; **d** postoperative correlation in individuals with insulin sensitivity. *p*-value less than 0.05 are considered statistically significant

FetA was significantly reduced during both the preoperative diet and during the six-weeks following surgery (Fig. 3). At baseline, average FetA was 606.1 ± 170.0μg/mL. Although there was no difference in FetA by sex (*p* = 0.75), older age was associated with lower baseline FetA in women only (*r* = − 0.52, *p* = 0.004). Baseline FetA was not significantly correlated with baseline HOMA-IR, BMI, % BF or insulin resistance status. Following the hypocaloric, preoperative diet, FetA declined to 533.3 ± 98.8μg/mL. Change in FetA was associated with baseline FetA, in that; those with the highest baseline FetA had the greatest reductions in FetA during the preoperative diet (*r* = − 0.83, *p* < 0.0001). In individuals with insulin resistance, preoperative FetA change was significantly correlated with preoperative BMI change (*r* = 0.58, *p* = 0.002), but not with % BF change, EBW loss or time (days) between T0 and T1. In individuals with insulin sensitivity, preoperative FetA change was not significantly associated with any of the aforementioned weight variables (Fig. 4, a-b).

**Fig. 5:**
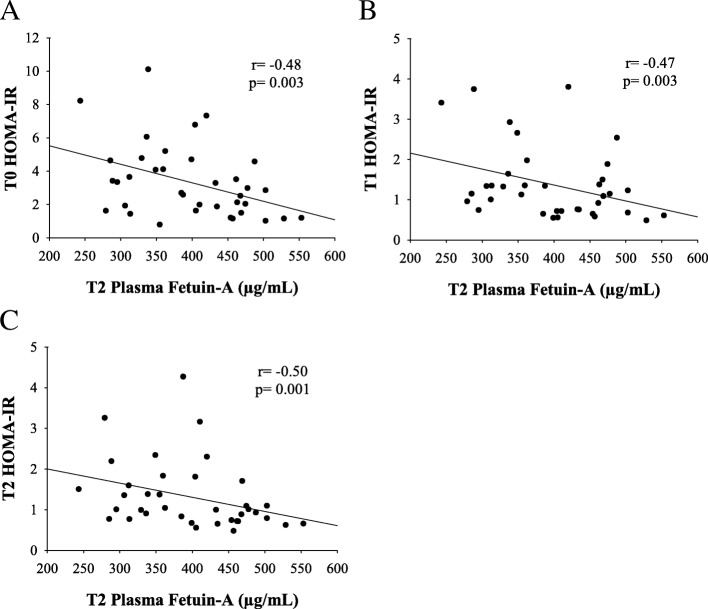
Correlation of postoperative Fetuin-A with HOMA-IR; **a** at baseline; **b** on the morning of surgery; **c** at six-week follow-up

Following surgery, FetA further decreased to 392.4 ± 82.9μg/mL. In individuals with insulin resistance, this change was related to BMI change (*r* = 0.42, *p* = 0.03) but not excess body weight loss or % BF change. Again, change in FetA in individuals with insulin sensitivity was not related to change in body composition (Fig. 4, c-d). FetA changes during the postoperative period were correlated with FetA changes during the preoperative diet (*r* = − 0.53, *p* = 0.0005). Additionally, the rate of FetA reduction (total FetA change divided by the time interval in days) was not significantly different during the pre- and postoperative periods. FetA and HOMA-IR were not associated at T0 or T1. However, HOMA-IR at T0, T1 and T2 were significantly associated with FetA at T2 (*p* = 0.003, 0.003 and 0.001, respectively) (Table 3, Fig. 5). Baseline FetA was not associated with reported caloric intake or macronutrient distribution at baseline. Change in FetA during the preoperative and postoperative diet was also not associated with caloric or macronutrient intake change during these times.

**Table 3:** Correlation of HOMA-IR and Fetuin-A by visit

Variable		Fetuin-A		
		T0	T1	T2
HOMA-IR	T0	*r* = − 0.27	*r* = − 0.13	*r* = − 0.48
		*p* = 0.111	*p* = 0.427	*p* = 0.003
	T1	*r* = − 0.33	*r* = − 0.23	*r* = − 0.47
		*p* = 0.044	*p* = 0.168	*p* = 0.003
	T2	*r* = − 0.30	*r* = − 0.14	*r* = − 0.50
		*p* = 0.068	*p* = 0.420	*p* = 0.001

Associations were evaluated using Pearson Correlation Coefficient. *p*-value less than 0.05 are considered statistically significant

### Fetuin-a and adipocyte size

**Fig. 6:**
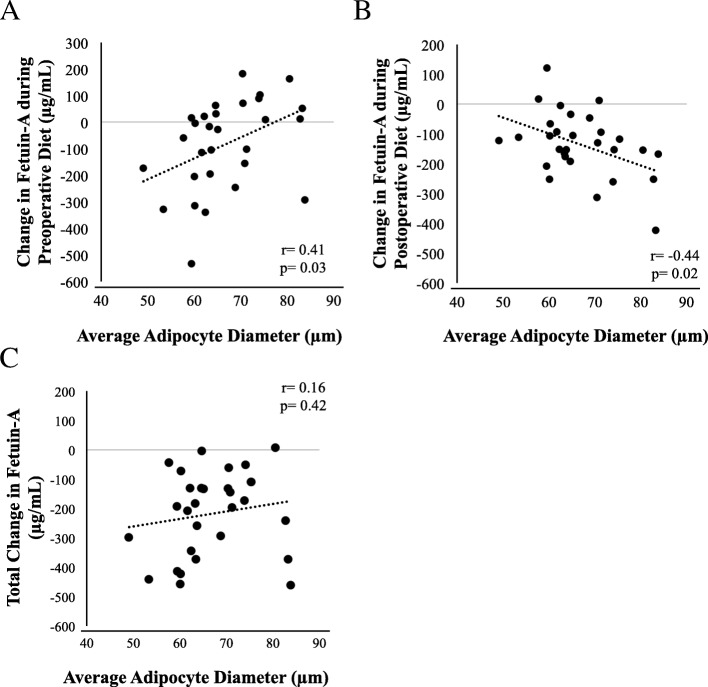
Association of adipocyte size with preoperative, postoperative and total change in Fetuin-A (*n* = 29). Adipocyte size on the day of surgery was significantly correlated with; **a**. change in preoperative FetA and **b**. change in postoperative FetA. **c**. No correlation was found between adipocyte diameter and total change in FetA. *p*-value less than 0.05 are considered statistically significant

Adipocyte diameter on the day of surgery did not differ by sex or age and was not associated with HOMA-IR or plasma FetA at T1. Adipocyte diameter tended to be larger in individuals with insulin resistance than those with insulin sensitivity (69.2 ± 8.2 vs. 62.9 ± 8.7; *p* = 0.060) although this did not reach statistical significance. Adipocyte size was correlated with change in HOMA-IR during the preoperative diet (*r* = − 0.38, *p* = 0.04) but not with change in BMI or % BF change. However, adipocyte diameter was correlated with change in FetA during the preoperative diet (*r* = 0.41, *p* = 0.03). In that, greater FetA reduction during the preoperative diet was associated with smaller adipocytes on the day of surgery (Fig. 6, a). Postoperatively, the inverse association was observed between FetA and adipocyte diameter (*r* = − 0.44, *p* = 0.02). Those with larger adipocytes on the day of surgery had greater postoperative change in FetA (Fig. 6, b). No association was found between total FetA change and adipose diameter (Fig. 6, c).

## Discussion

The preoperative diet regimen offers an important period for studying how individuals with morbid obesity respond to acute dietary changes prior to receiving weight loss surgery. However, the literature often fails to account for this time period. In the case of FetA, we found that FetA was significantly reduced following the preoperative diet and during the weeks immediately following SG. Although the time period during the preoperative diet and the postoperative period differed (2 weeks vs. 6 weeks), the rate of FetA change was not significantly different. Factors associated with FetA change differed by insulin sensitivity status. Those with insulin resistance had stronger correlations between FetA change and BMI change than individuals with insulin sensitivity. Caloric intake was significantly reduced over the course of this study. However, change in calories and macronutrient distribution did not explain changes in circulating FetA. Notably, the conclusions drawn in this study rely on self-reported food logs. Controlled feeding studies in human subjects would improve our knowledge of dietary factors which influence FetA.

In contrast to previous reports, we did not find a consistent association between FetA and HOMA-IR during each visit. One possible explanation is that previous studies completed cross-sectional analysis of samples, while our cohort was actively attempting weight loss and experiencing fluctuations in dietary intake. Thus, we also explored whether FetA may be correlated with HOMA-IR at other time points. We found that that HOMA-IR at T0, T1 and T2 was significantly associated with FetA at T2. Specifically, higher HOMA-IR at any time point predicted lower FetA postoperatively suggesting that the greatest FetA improvements occur in those with insulin resistance.

Adipocyte size has been associated with insulin resistance, therefore, we next evaluated whether circulating FetA was associated with omental adipocyte size. Greater reduction in FetA before SG was associated with smaller adipocytes on the day of surgery while reported changes in dietary intake, % BF loss or BMI loss were not associated with adipocyte size. The mechanistic link between FetA and adipocyte size is not entirely clear. One possible explanation is the relationship of FetA with PPAR-γ. PPAR-γ is a nuclear receptor found in the adipose, which plays an important role in adipocyte differentiation and lipid metabolism [23]. PPAR-γ is stimulated by ligands including TZDs and fatty acids [24]. Activated PPAR-γ increases transcription of proteins such as adiponectin, lipoprotein lipase and adipocyte binding protein and decreases cytokines such as tumor necrosis factor alpha and interleukin-6 [25–28]. Thus, PPAR-γ activation is associated with greater lipid uptake and adipogenesis [29, 30]. PPAR-γ activity is inhibited via Wnt signaling. For adipogenesis to occur, suppression of Wnt signaling is essential [31]. Agarwal et al. recently demonstrated that FetA upregulates Wnt3A thus decreasing PPAR-γ [30]. Therefore, greater reductions in FetA may relieve suppression of PPAR-γ, allow for hyperplasia and smaller adipocytes. However, if FetA remains elevated, Wnt-suppression of PPAR-γ may lead to adipocyte hypertrophy and unopposed proinflammatory cytokine release [28, 32]. Our data support this hypothesis because we found that greater reductions in FetA preoperatively were correlated with smaller adipocytes on the day of surgery. The opposite correlation was seen postoperatively. Specifically, those with larger adipocytes on the day of surgery, had larger reductions in FetA in the 6-weeks following surgery. This supports that change in circulating FetA may correlate with adipocyte size.

Because omental adipocyte diameter was only measured at one time point during this study, it is also possible that change in adipocyte size influences circulating FetA via alternative pathways. For example, activation of PPAR-γ via TZDs increases insulin sensitivity and reduces FetA expression in liver [33]. Thus, the directionality of this relationship remains unclear and warrants further investigation. Additional studies, such as adipose-specific knockout of FetA, are needed to determine the influence of adipocyte-derived FetA on adipocyte size and insulin function. An additional limitation of this study is that tissue was collected and processed for the measurement of adipocyte diameter via histological analysis. More complex analysis of adipocyte volume and distribution would likely yield more informative insights about the interaction of FetA and adipocyte size.

The present study is unable to determine whether the decrease in circulating FetA after bariatric procedures is due to a reduction in adipose- or liver-derived FetA. While the liver is recognized as the main site for FetA production, adipose expression of FetA does appear to be related to adiposity status. Adipose from obese adults with T2DM had three-times higher FetA expression than adipose tissue from normal weight humans without T2DM [15]. Obese rats had increased FetA expression in visceral adipose compared to lean rats and the expression of FetA in omental fat was reduced by fasting [34]. Thus, in states of morbid obesity, the adipose may be an important contributor to circulating FetA and weight loss and calorie restriction may lead to reduced expression of FetA from the adipocyte.

## Conclusions

Our results suggest that FetA, a marker of relevance to T2DM which quickly improves following bariatric surgery, actually begins to improve preoperatively during calorie restriction. Greater preoperative FetA reduction was associated with smaller omental adipocytes on the day of surgery. This relationship has not been reported previously in the literature and may offer insight into the relationship between FetA and adipocyte function. Furthermore, this study highlighted the importance of monitoring changes which occur during the preoperative diet when evaluating acute bariatric outcomes, such as improvements in glucose homeostasis. The preoperative dietary protocol not only significantly reduced FetA but also substantially decreased HOMA-IR. Because uncontrolled hyperglycemia is a modifiable risk factor for multiple, non-bariatric procedures, this dietary protocol may have potential for broader use to reduce risk in patients preparing for procedures beyond bariatric surgery.

## Abbreviations

% BF: Percent Body Fat
BMI: Body Mass Index
DOS: Day of Surgery
EBW: Excess Body Weight
FetA: Fetuin-A
HOMA-IR: Homeostatic Model Assessment for Insulin Resistance
I-OWLS: Individual Outcomes of Weight Loss Surgery
PPAR-γ: Peroxisome Proliferator-Activated Receptor Gamma
RYGB: Roux-en-Y Gastric Bypass
SG: Sleeve Gastrectomy
T2DM: Type 2 Diabetes Mellitus
TZDs: Thiazolidinediones
UIUC: University of Illinois at Urbana-Champaign
